# Death of Dr. Blackburn

**Published:** 1897-12

**Authors:** 


Death of Dr. Blackburn.—Died, at Dallas, Texas, November 23, 1897, at the home of his son-in-law, Judge Muse, Dr. J. H. Blackburn, late of Mineral Wells, Texas, where he took up his residence some years ago, for the benefit of the waters, he being a sufferer from organic kidney disease. Dr. Blackburn graduated at the New Orleans School of Medicine in 1861, and settled in Brenham, Texas, where he practiced a good many years.

